# Psilocybin-Assisted Therapy in Psychiatry: A Narrative Clinical Review of Mechanisms, Therapeutic Applications, and Emerging Evidence (2025–2026)

**DOI:** 10.3390/jcm15166228

**Published:** 2026-08-12

**Authors:** Teodora Anghel, Adriana Cojocaru, Lavinia Hogea, Iuliana Costea, Amalia Marinca, Raluca Dumache, Laura Nussbaum, Iuliana-Anamaria Trăilă

**Affiliations:** 1 Neuroscience Department, “Victor Babes” University of Medicine and Pharmacy, 300041 Timisoara, Romania; anghel.teodora@umft.ro; 2 Neuropsychology and Behavioral Medicine Center, “Victor Babes” University of Medicine and Pharmacy, 300041 Timisoara, Romania; raluca.dumache@umft.ro (R.D.); nussbaum.laura@umft.ro (L.N.); 3 Psychology Department, West University of Timisoara, 300223 Timisoara, Romania; iuliana.costea@e-uvt.ro; 4 Center for Studies and Research in Psychology, Faculty of Psychology, “Tibiscus” University, Lascăr Catargiu 4-6, 300559 Timisoara, Romania; amalia_marinca@yahoo.com; 5 Ph.D. School, Faculty of Medicine, Department of Microscopic Morphology, Genetics Discipline, Center of Genomic Medicine, “Victor Babes” University of Medicine and Pharmacy, 300041 Timisoara, Romania; iuliana.traila@umft.ro

**Keywords:** psilocybin, psychedelic-assisted therapy, major depressive disorder, treatment-resistant depression, neuroplasticity, substance use disorders, altered states, mystical experience, 5-HT2A receptor, veterans, PTSD

## Abstract

**Background/Objectives:** Psilocybin-assisted therapy has progressed from early proof-of-concept work to a substantially expanded evidence base, with four pivotal studies published in 2025–2026 that were not incorporated into earlier reviews. This review synthesizes the pharmacological and neurobiological foundations of psilocybin and appraises clinical evidence across major depressive disorder (MDD), treatment-resistant depression (TRD), post-traumatic stress disorder (PTSD), cancer-related distress, and substance use disorders, emphasizing long-term durability, expanding indications, and methodological limitations. **Methods:** A narrative review was conducted using a targeted search of PubMed/MEDLINE, Embase, Scopus, and Web of Science (January 2000–April 2026), emphasizing 2025–2026 publications. Predefined eligibility principles prioritized randomized controlled trials, long-term follow-up studies, and systematic reviews; formal PRISMA procedures were not applied, consistent with the narrative design. **Results:** Four 2025–2026 studies extend the evidence base: a 52-week follow-up confirming dose-dependent maintenance of antidepressant benefit after a single 25 mg psilocybin session; the first pilot study in Veterans with severe TRD, reporting a 60% response rate at three weeks; the first safety trial in PTSD, where symptom improvement tracked self-transcendent experience intensity but worsened with session anxiety; and a living review of 15 randomized trials confirming a meaningful antidepressant effect while identifying functional unblinding as a substantial threat to effect estimates. **Conclusions**: Evidence supports psilocybin-assisted therapy as mechanistically distinct and clinically promising, most strongly in MDD and TRD, with preliminary support in PTSD and Veterans. Functional unblinding, small open-label designs, narrow safety populations, and absent SSRI-integration protocols constrain the current conclusions.

## 1. Introduction

Psilocybin (4-phosphoryloxy-N,N-dimethyltryptamine) is a psychedelic tryptamine found in over 200 fungal species, primarily of the genera *Psilocybe* and *Stropharia*. Upon oral ingestion, it undergoes rapid dephosphorylation to psilocin, which acts on the central nervous system principally through partial agonism at 5-HT2A serotonin receptors [1]. Clinical investigation of psilocybin between 1950 and 1966 generated a substantial body of preliminary literature [2], but scheduling under the 1971 United Nations Convention on Psychotropic Substances halted formal clinical research for nearly three decades. Investigation resumed in the late 1990s, beginning with Vollenweider et al.’s 1997 neuroimaging work distinguishing psilocybin’s metabolic signature from that of schizophrenia, and has expanded substantially over the past two decades [3].

By 2025, psilocybin holds FDA Breakthrough Therapy Designation for treatment-resistant depression (granted to COMPASS Pathways, 2018) and for major depressive disorder (granted to the Usona Institute, 2019). Treatment-resistant depression (TRD) remains one of the greatest unmet needs in contemporary psychiatry, affecting approximately one-third of patients with major depressive disorder and being associated with substantial functional impairment, increased healthcare utilization, elevated suicide risk, and considerable socioeconomic burden. The limited effectiveness of conventional monoaminergic antidepressants in this population has driven the search for mechanistically distinct therapeutic approaches capable of targeting alternative neurobiological pathways involved in depression pathophysiology [4]. Regulatory pathways for restricted clinical access have also emerged outside formal drug approval: since 1 July 2023, Australia’s Therapeutic Goods Administration permits psilocybin prescribing by specifically authorized psychiatrists for treatment-resistant depression only, under the Authorized Prescriber Scheme [5]; Oregon and Colorado have established state-regulated, non-medical psilocybin services frameworks operating outside the FDA approval pathway [6,7]. None of these pathways constitutes FDA marketing approval, and clinical translation outside trial settings remains limited and jurisdiction-specific.

Four studies published in 2025 substantially extend this evidence base: a 52-week long-term observational follow-up of the COMPASS Pathways phase 2 trial [8], the first open-label study of psilocybin in U.S. military Veterans with severe treatment-resistant depression [9], the first dedicated safety and feasibility trial of psilocybin in PTSD [10], and a living systematic review and meta-analysis of 15 RCTs [1]. This review synthesizes these findings with the existing literature to assess the current state of the evidence base.

Despite rapidly expanding evidence, uncertainties remain regarding long-term efficacy, generalizability, safety across diverse clinical populations, and the integration of psilocybin-assisted therapy into routine psychiatric care.

This narrative review aims to: (i) synthesize the historical, ethnobotanical, and pharmacological foundations of psilocybin; (ii) critically appraise evidence from randomized controlled trials and systematic reviews published through 2025, with particular emphasis on four studies published in 2025 that substantially extend earlier syntheses of this literature (a 52-week long-term follow-up of the COMPASS Pathways phase 2 trial, the first open-label study in military Veterans with severe treatment-resistant depression, the first dedicated safety trial in PTSD, and a living systematic meta-analysis of 15 RCTs); (iii) examine the neurobiological mechanisms underlying psilocybin’s therapeutic effects, including 5-HT2A receptor-mediated neuroplasticity, BDNF/TrkB signalling, and Default Mode Network modulation; (iv) evaluate evidence for psilocybin’s effects in psychedelic-naive healthy populations; and (v) contextualize these findings within the current regulatory and clinical-integration landscape.

## 2. Materials and Methods

### 2.1. Review Design

This study was conducted as a narrative clinical review aimed at synthesizing contemporary evidence regarding psilocybin-assisted therapy in psychiatric disorders. Given the rapidly evolving nature of the field, a narrative approach was selected to integrate findings from randomized controlled trials, longitudinal follow-up studies, systematic reviews, meta-analyses, neurobiological investigations, regulatory documents, and consensus statements. The review emphasizes clinical applicability, translational relevance, and emerging evidence published between 2025 and 2026, while critically discussing methodological limitations and implementation challenges in routine psychiatric practice.

### 2.2. Literature Search Strategy

A comprehensive literature search was conducted in PubMed/MEDLINE, Embase, Scopus, and Web of Science to identify relevant publications on psilocybin-assisted therapy and psychiatric disorders. The search covered studies published from January 2000 to April 2026, with particular emphasis on evidence generated between 2020 and 2026 and on pivotal reports published during 2025–2026.

The search strategy combined controlled vocabulary and free-text terms related to psilocybin and mental health conditions, including: “psilocybin”, “psilocybin-assisted therapy”, “psychedelic-assisted psychotherapy”, “major depressive disorder”, “treatment-resistant depression”, “post-traumatic stress disorder”, “substance use disorder”, “alcohol use disorder”, “tobacco dependence”, “neuroplasticity”, “5-HT2A receptor”, and “default mode network”. Boolean operators (“AND”, “OR”) were applied as appropriate to optimize retrieval.

Additional sources included reference lists of relevant articles, recent consensus statements, regulatory documents issued by the U.S. Food and Drug Administration and other national authorities, and landmark publications from major clinical research groups, including Johns Hopkins University, Imperial College London, New York University, and the COMPASS Pathways clinical program.

### 2.3. Study Selection and Eligibility Criteria

Owing to the narrative nature of this review, formal systematic review procedures and PRISMA reporting guidelines were not applied. Nevertheless, predefined eligibility principles were employed to enhance transparency and minimize selection bias.

Study identification, screening, and eligibility assessment were performed independently by two reviewers using the predefined eligibility principles described above. Any discrepancies regarding study inclusion were resolved through discussion until consensus was reached.

Priority was given to randomized controlled trials, prospective cohort studies, long-term follow-up investigations, systematic reviews, meta-analyses, and high-impact mechanistic studies addressing the therapeutic applications, safety profile, and neurobiological effects of psilocybin. Regulatory documents, expert consensus statements, and policy reports were included when directly relevant to clinical implementation.

Publications not written in English, conference abstracts lacking peer-reviewed full texts, duplicate reports, and studies focused exclusively on recreational or non-clinical use without therapeutic implications were excluded from detailed analysis. When multiple reports described the same clinical cohort, the most recent and comprehensive publication was preferentially considered.

### 2.4. Data Extraction and Narrative Synthesis

Relevant information was extracted regarding study design, patient populations, psychiatric indications, dosing protocols, psychotherapeutic support models, efficacy outcomes, safety findings, neurobiological mechanisms, and long-term follow-up data. Particular attention was devoted to studies published in 2025, including investigations involving treatment-resistant depression, military veterans, post-traumatic stress disorder, and updated evidence syntheses.

The evidence was synthesized narratively and organized into thematic domains encompassing historical development, pharmacology, neurobiology, clinical applications, emerging evidence, safety considerations, regulatory frameworks, and future directions. Methodological limitations, including functional unblinding, expectancy effects, restricted external validity, and implementation challenges, were critically examined throughout the review.

## 3. Historical Background and the Evolution of Psilocybin Research

### 3.1. Indigenous and Traditional Use

Archaeological and ethnobotanical evidence suggests that psychoactive mushrooms have been used in ritual and medicinal contexts for millennia. Indigenous cultures in Mesoamerica, particularly the Aztec and Maya civilizations, referred to these fungi as Teonanácatl (“flesh of the gods”) and used them in ceremonial practices for healing and spiritual transformation [11]. Although the precise historical continuity of these traditions remains debated, they provide important cultural context for contemporary therapeutic applications.

### 3.2. Modern Scientific Development

Modern scientific investigation of psilocybin began with its isolation and synthesis by Albert Hofmann in 1958 [12]. Although international scheduling in 1971 substantially restricted clinical research for several decades, renewed investigations in the 1990s and early 2000s laid the foundation for contemporary psychedelic science. Seminal contributions from research groups at the University of Zurich, Johns Hopkins University, Imperial College London, New York University, and the Heffter Research Institute established the initial evidence supporting the therapeutic potential of psilocybin-assisted interventions across multiple psychiatric disorders [3].

## 4. Pharmacology and Neurobiological Mechanisms

### 4.1. Pharmacokinetics and Pharmacodynamics

Psilocybin is a prodrug belonging to the tryptamine class. Following oral ingestion, alkaline phosphatases rapidly dephosphorylate it to psilocin, with peak plasma concentrations reached within 1.8–4 h and oral bioavailability of approximately 53% [13]. Hepatic biotransformation proceeds via cytochrome P450 (CYP)-mediated oxidation—predominantly CYP2D6, with a secondary CYP3A4 contribution of approximately 40%—alongside monoamine oxidase A activity and, as the dominant pathway in vivo, UGT1A9/UGT1A10-mediated glucuronidation to psilocin-O-glucuronide, the major circulating metabolite [13,14]. Renal excretion clears psilocin and its conjugates, with a reported elimination half-life of approximately 1.5–4 h, though estimates vary by assay and sampling schedule [13]. CYP2D6-dependent metabolism raises a theoretical, as-yet uncharacterized concern for altered exposure in poor metabolizers, relevant to future personalized dosing protocols.

Concomitant SSRIs/SNRIs reliably attenuate the subjective psychedelic response, attributed to 5-HT2A receptor downregulation under chronic serotonergic tone; the theoretical risk of serotonin syndrome remains clinically unsubstantiated, and a phase II trial of psilocybin as an SSRI-adjunct in TRD reported an acceptable safety profile [15]. Nonetheless, most psilocybin RCTs require an SSRI/SNRI washout period of two to four weeks before enrolment [16], meaning that current evidence predominantly reflects monotherapy rather than real-world augmentation—a translational gap directly relevant to this Special Issue’s focus on combination strategies.

Acute cardiovascular effects warrant explicit monitoring, with meta-analytic data confirming that blood pressure fluctuations are significantly more frequent as an adverse event than with a comparator [16]. QTc prolongation is generally modest, below the 10 ms threshold of regulatory concern. Chronic or repeated low-dose exposure raises a theoretical risk of 5-HT2B-mediated cardiac valvulopathy, a mechanism established for other serotonergic agents, warranting echocardiographic surveillance in repeated-dosing protocols. Current trials conservatively exclude baseline blood pressure ≥ 140/90 mmHg, though recent pooled analyses question whether this threshold is unduly restrictive [17].

### 4.2. Serotonergic Signaling and Receptor Mechanisms

Psilocin acts as a partial agonist with the highest affinity at 5-HT2A, with secondary affinity at 5-HT2B, moderate affinity at 5-HT2C, and lower affinity at 5-HT1A and 5-HT1D [1,18]. The 5-HT2A receptor, densely expressed on neocortical pyramidal neurons, is necessary and central to psilocybin’s psychoactive and antidepressant effects: pharmacological blockade abolishes the subjective response [18]. Co-localization of 5-HT2A and 5-HT1A on GABAergic interneurons shapes the excitatory-inhibitory balance underlying subjective effects, including ego dissolution, while 5-HT2C activation appears to exert an opposing, dopamine-suppressing influence relative to 5-HT2A. Notably, preclinical work indicates psilocin’s antidepressant-like behavioral and synaptic actions can occur independently of 5-HT2A activation under certain experimental conditions [19], suggesting the receptor pharmacology underlying acute psychedelic effects and that underlying sustained antidepressant benefit may be partially dissociable. Downstream, 5-HT2A signaling recruits β-arrestin and phospholipase C pathways and couples to intracellular BDNF/TrkB and mTOR signaling [20,21], pathways associated with structural synaptic remodeling rather than acute neurotransmission.

### 4.3. Default Mode Network Modulation

The Default Mode Network (DMN) (comprising the medial prefrontal cortex, posterior cingulate cortex, and angular gyrus) is associated with self-referential processing, ruminative cognition, and mind-wandering. DMN hyperactivity is a well-established neuroimaging correlate of major depression [22]. Psilocybin reliably decreases DMN functional connectivity and cerebral blood flow acutely [23,24], potentially reducing maladaptive self-referential cognition and rumination. Post-treatment fMRI demonstrates that acute DMN suppression is followed by a sustained increase in global brain network integration [25], which correlates directly with antidepressant response.

### 4.4. Neuroplasticity, BDNF, and TrkB Signalling

A landmark 2023 paper in Science demonstrated that psilocybin promotes structural neuroplasticity by activating intracellular 5-HT2A receptors, thereby stimulating dendritic arbor complexity and spinogenesis independently of extracellular serotonin [20]. Zhao et al. demonstrated in rodent models that a single psilocybin dose produced rapid, sustained antidepressant-like effects accompanied by increased dendritic spine density and complexity in the prefrontal cortex and hippocampus, elevated synaptic protein levels (p-GluA1, PSD95, synapsin-1), and activation of BDNF/TrkB and mTOR signaling—effects persisting weeks after a single dose [21]. These findings suggest that psilocybin may engage neuroplastic processes through mechanisms that differ from those traditionally associated with chronic monoaminergic treatments. A 2023 systematic review in Neuropsychopharmacology identified at least two pharmacologically distinct plasticity mechanisms: 5-HT2A-mediated and TrkB-mediated pathways [26].

Beyond its effects on neuroplasticity, accumulating evidence suggests that psilocybin may also exert immunomodulatory and anti-inflammatory effects, representing an additional mechanism that could contribute to its therapeutic potential. Experimental studies have demonstrated that psilocybin and psilocin reduce the production of pro-inflammatory cytokines and modulate immune-cell responses, effects that appear to be at least partially independent of the subjective psychedelic experience. Notably, anti-inflammatory activity has been observed even under conditions of 5-HT2A receptor blockade, suggesting that these actions are not solely mediated through the canonical serotonergic pathway responsible for the acute psychoactive effects. Although the precise molecular mechanisms remain to be fully elucidated, these findings broaden the current understanding of psilocybin’s pharmacology and highlight inflammation as a promising target for future translational and clinical research, particularly in psychiatric disorders increasingly recognized to involve immune dysregulation [27].

## 5. Therapeutic Framework and Subjective Experience

### 5.1. Set, Setting, and Psychotherapeutic Integration

Contemporary protocols incorporate comprehensive psychiatric assessment, preparatory psychotherapy, supervised dosing sessions, and post-session integration, emphasizing patient safety and therapeutic alliance [28]. Screening typically includes a structured psychiatric interview, medical history, and assessment of personal and family psychiatric history to exclude high-risk profiles (Section 5.3). Written informed consent addresses the unpredictable and potentially intense nature of the subjective experience prior to enrolment. Preparatory sessions establish therapeutic rapport and psychoeducation; the dosing session itself (typically 6–8 h, with eye shades and curated music) is conducted under continuous monitoring by trained clinical staff, including periodic vital sign assessment given the cardiovascular effects discussed in Section 4.1; and structured integration sessions follow to support meaning-making and consolidate clinical gains.

### 5.2. Mystical-Type Experiences and Ego Dissolution

Higher doses (≥20 mg) are associated with marked alterations in self-processing, sometimes described as ego dissolution—a transient attenuation of the boundary between self and environment [28]. These self-transcendent experiences, characterized by changes in autobiographical perspective and altered self-referential processing, are the strongest predictor of long-term therapeutic benefit across indications, including depression [29,30], cancer-related distress [31,32], and personality change [33]. Mechanistically, this phenomenology is consistent with the acute Default Mode Network suppression described in Section 4.3. Importantly, the identification of neurobiological correlates does not diminish the potential therapeutic importance of the subjective psychological experience itself. Rather, current evidence suggests that neurobiological changes and psychologically meaningful shifts in self-perception, autobiographical perspective, and personal meaning are likely to interact in producing the clinical effects of psilocybin [10].

### 5.3. Challenging Experiences and Safety Considerations

Challenging experiences (‘bad trips’), typically involving transient anxiety, fear, or dysphoria, occur in a subset of participants; over 75% of those reporting such experiences nonetheless rate them as personally meaningful or beneficial in retrospect [34]. Acute adverse effects most frequently reported in clinical trials include headache, nausea, anxiety, dizziness, and transient elevations in blood pressure and heart rate; the pharmacology of these effects is detailed in Section 4.1 [35]. Contemporary protocols substantially mitigate risk through standardized exclusion criteria, which across published trial protocols consistently exclude: personal or first-degree family history of psychotic disorders, schizophrenia-spectrum disorders, or bipolar I disorder; active suicidal ideation with intent or plan; uncontrolled hypertension, unstable cardiac disease, or recent cardiovascular events; and current substance use disorder other than mild alcohol or tobacco use. The evidentiary basis for blanket family-history exclusions, particularly for bipolar disorder, has been increasingly questioned as overly conservative and lacking systematic safety data, with recent proposals favoring graded risk-stratification over categorical exclusion—a consideration directly relevant to expanding eligible populations as the field matures. Suicidality monitoring remains a standard component of trial protocols irrespective of indication, consistent with practice in the COMP 001 trial [36]; no causal relationship between psilocybin administration and completed suicide has been established in any clinical study to date.

## 6. Clinical Applications of Psilocybin-Assisted Therapy

### 6.1. Clinical Evidence and Long-Term Therapeutic Outcomes

#### 6.1.1. Foundational Clinical Trials (2016–2023)

Early clinical investigations established the feasibility and therapeutic potential of psilocybin-assisted therapy in patients with major depressive disorder (MDD) and treatment-resistant depression (TRD). In the first modern open-label study, Carhart-Harris et al. reported response and remission rates of 67% and 42%, respectively, one week after treatment, with sustained benefits observed in a subset of patients at three months [24]. Subsequent randomized controlled trials further strengthened this evidence. The landmark New England Journal of Medicine study comparing psilocybin with escitalopram demonstrated comparable improvements in the primary outcome and superior effects across several secondary measures, including emotional well-being and anhedonia [37]. Similarly, Davis et al. reported response and remission rates of 71% and 58%, respectively, four weeks after treatment in patients with MDD [30].

Larger multicenter investigations have subsequently confirmed both efficacy and acceptable tolerability. Goodwin et al. identified the 25 mg dose as the most effective therapeutic regimen in patients with TRD across 22 international sites [36], while Raison et al. demonstrated rapid antidepressant effects occurring within eight days of administration [38]. Collectively, these studies established the foundation for ongoing phase III programs and expanded interest in psilocybin as a novel therapeutic strategy for depressive disorders.

#### 6.1.2. Long-Term Outcomes and Emerging Evidence (2025–2026)

Long-term durability remains a critical consideration in evaluating the clinical utility of psilocybin-assisted therapy. The COMP004 observational follow-up study monitored participants from the COMP001 trial for 52 weeks and demonstrated prolonged maintenance of antidepressant benefits among individuals receiving a single 25 mg dose of COMP360 compared with lower-dose groups [8]. Importantly, most adverse events were mild or moderate, supporting the overall tolerability of the intervention while emphasizing the need for larger confirmatory studies.

Emerging evidence has also extended investigation to highly treatment-resistant populations. Ellis et al. reported clinically meaningful improvements in depressive symptoms among military veterans with severe TRD, with therapeutic effects persisting up to 12 months after treatment in a substantial proportion of participants [9]. Although limited by its open-label design and small sample size, this study suggests that psilocybin-assisted therapy may retain efficacy even in patients with extensive prior treatment failures and psychiatric comorbidity.

#### 6.1.3. Clinical Implications and Remaining Challenges

The available evidence indicates that psilocybin-assisted therapy may offer rapid and sustained symptom improvement in selected patients with MDD and TRD. Nevertheless, important challenges remain before widespread clinical implementation can be considered. Functional unblinding, expectancy effects, highly selected study populations, and limited long-term safety data continue to constrain interpretation of current findings and may influence estimates of therapeutic efficacy [10].

Furthermore, unresolved questions persist regarding optimal maintenance strategies, integration with conventional antidepressant treatments, therapist training requirements, and cost-effectiveness in real-world settings. Ongoing phase III trials and larger pragmatic studies will be essential to define the precise role of psilocybin-assisted therapy within contemporary psychiatric practice and to establish evidence-based recommendations for patient selection and clinical monitoring.

### 6.2. Post-Traumatic Stress Disorder

Post-traumatic stress disorder (PTSD) affects approximately 3.9% of the global population and remains associated with substantial psychiatric morbidity, impaired quality of life, and incomplete responses to existing pharmacological and psychotherapeutic interventions [3]. Growing interest in psychedelic-assisted therapy has prompted investigations into psilocybin as a potential adjunctive treatment, particularly for patients with complex trauma histories and treatment resistance.

McGowan et al. (2025) conducted the first dedicated open-label clinical study evaluating the safety and preliminary efficacy of a single 25 mg dose of COMP360 in patients with PTSD [10]. Psilocybin was generally well tolerated, and improvements in CAPS-5 scores were positively associated with the intensity of self-transcendent experiences during treatment, whereas heightened anxiety and emotional distress predicted less favorable outcomes. Despite these encouraging findings, the absence of randomized controls, small sample sizes, and limited long-term follow-up underscores the need for adequately powered trials to establish efficacy, optimal patient selection criteria, and disorder-specific therapeutic protocols.

### 6.3. Cancer-Related Psychological and Existential Distress

Psychological distress, depression, and existential suffering are common among patients with life-threatening malignancies and significantly impair quality of life and treatment outcomes [39]. In a randomized controlled trial, Ross et al. demonstrated that a single dose of psilocybin produced substantial and sustained reductions in anxiety, depression, and hopelessness among patients with advanced cancer, with benefits persisting at 26 weeks. Similarly, Griffiths et al. reported clinically meaningful improvements in mood and anxiety symptoms in approximately 80% of participants, with remission rates approaching 60% at six-month follow-up [31].

Long-term observations further support the durability of these effects. Agin-Liebes et al. documented sustained psychological benefits 4.5 years after treatment, with 71–80% of participants maintaining clinically significant improvements [40]. Although larger confirmatory studies are required, these findings suggest that psilocybin-assisted therapy may represent a valuable adjunct within psycho-oncology, particularly for addressing existential distress and enhancing psychological adaptation to severe illness.

### 6.4. Substance Use Disorders

#### 6.4.1. Tobacco Use Disorder

Initial evidence supporting psilocybin-assisted therapy for tobacco dependence emerged from the pilot study conducted by Johnson et al., which reported biochemically verified abstinence rates of 80% at six months among long-term smokers receiving psilocybin in combination with structured behavioral support [41]. Five-year follow-up data demonstrated sustained abstinence in 60% of participants, suggesting the possibility of durable behavioral change following limited therapeutic exposure.

The mechanisms underlying these effects remain incompletely understood but likely involve enhanced cognitive flexibility, reduced maladaptive reward processing, and profound shifts in self-perception and motivation. The integration of psychologically meaningful experiences with established behavioral interventions may contribute to long-term smoking cessation, although larger randomized trials remain necessary to validate these preliminary findings [41].

#### 6.4.2. Alcohol Use Disorder

Psilocybin-assisted therapy has also shown promise in alcohol use disorder (AUD). In the first randomized clinical trial addressing this indication, Bogenschutz et al. demonstrated significantly fewer heavy-drinking days among patients receiving psilocybin-assisted psychotherapy compared with active placebo during a 32-week follow-up period [42]. More recently, a Swiss feasibility trial reported prolonged abstinence and improvements in depressive symptoms following a single psilocybin session combined with brief psychotherapeutic support [43].

A systematic review published in 2023 further highlighted consistent therapeutic signals across studies investigating psychedelic-assisted interventions in addiction medicine [44]. Nevertheless, the current evidence base remains limited by relatively small cohorts, heterogeneous psychotherapeutic protocols, and challenges related to blinding and expectancy effects, emphasizing the need for larger multicenter studies and standardized treatment frameworks.

### 6.5. Safety, Tolerability, and Patient Selection

Across contemporary clinical trials, psilocybin has generally demonstrated an acceptable safety profile when administered within structured therapeutic settings and under appropriate medical supervision [45]. The most frequently reported adverse events include transient anxiety, headache, nausea, dizziness, fatigue, and temporary increases in blood pressure and heart rate. These effects are typically self-limited and resolve without long-term sequelae, while serious adverse reactions remain uncommon in carefully screened populations.

Comprehensive psychiatric assessment remains essential before treatment initiation. Current protocols generally exclude individuals with schizophrenia spectrum disorders, bipolar I disorder, active psychosis, or a strong family history of psychotic illness because of concerns regarding symptom exacerbation and altered vulnerability to psychedelic experiences [45]. Continuous monitoring for suicidality is also recommended, particularly among patients with severe treatment-resistant depression, given the underlying psychiatric burden and the limited availability of long-term safety data.

Potential pharmacological interactions warrant additional consideration. Concomitant use of serotonergic agents, including selective serotonin reuptake inhibitors and monoamine oxidase inhibitors, may alter therapeutic responses or modify subjective effects, although the clinical significance of these interactions remains incompletely understood. Standardized approaches to medication management before psilocybin administration have yet to be established and constitute an important area for future research.

Appropriate patient selection and therapeutic preparation are fundamental determinants of safety and efficacy. Contemporary treatment models incorporate comprehensive screening procedures, preparatory psychotherapy sessions, supervised dosing environments, and structured post-session integration. These components not only reduce the risk of adverse psychological reactions but also facilitate meaning-making processes that may contribute to sustained therapeutic benefits.

Finally, the implementation of psilocybin-assisted therapy in routine clinical practice will require multidisciplinary collaboration involving psychiatrists, psychologists, nurses, and other mental health professionals. The development of standardized training programs, monitoring protocols, and evidence-based clinical guidelines will be critical to ensuring safe, equitable, and effective delivery of this emerging therapeutic modality [45].

## 7. Discussion

The evidence reviewed in this article—anchored by four studies published in 2025–2026 that were unavailable to earlier syntheses of this literature [8,9,10,45]—indicates that psilocybin-assisted therapy has moved from early proof-of-concept work toward a more mature, though still incomplete, evidence base across several psychiatric indications. Four themes from this updated evidence warrant particular consideration, alongside the methodological constraints that continue to limit the strength of the conclusions that can be drawn.

Long-term durability: The COMP004 observational follow-up [46] addresses a question that had remained largely unanswered in this field: whether acute therapeutic benefits persist beyond the weeks-to-months horizon captured by most prior trials. The finding that a single 25 mg dose of COMP360 was associated with longer maintenance of antidepressant benefit at 52 weeks relative to the 1 mg and 10 mg comparator doses [46] is the first large-scale, dose-stratified evidence of this kind in psilocybin research and represents a meaningful addition to the literature. This finding should nonetheless be interpreted within the constraints of its design: COMP004 was an observational follow-up rather than a randomized, controlled extension, time-to-relapse was the primary endpoint rather than a directly measured maintenance of symptom remission, and the absence of a comparator arm receiving an active conventional antidepressant over the same 52-week window means the claim that this durability profile is superior to SSRI maintenance therapy cannot yet be made directly—it can only be inferred by analogy to the separate body of literature on antidepressant discontinuation. Direct, head-to-head comparative durability data remain a priority for Phase 3 trial design.

Expanding indications in treatment-resistant and high-burden populations: The pilot data in Veterans with severe TRD [46] and the safety trial in PTSD [47] extend the therapeutic hypothesis to populations defined by extensive prior treatment failure and, in many cases, psychiatric comorbidity. The 60% response rate reported by Ellis et al. at three weeks in Veterans who had failed an average of more than five prior treatments is a clinically notable signal in a population for whom few effective options exist; however, this finding derives from an open-label pilot study in 15 participants, with no control condition, and response rates of this magnitude reported in small, unblinded, single-arm studies have not infrequently failed to replicate at comparable effect sizes in subsequent controlled trials in psychiatric research more broadly. The finding should be read as hypothesis-generating rather than confirmatory. The PTSD findings [46] similarly address a population previously excluded from psychedelic trials on the theoretical concern that ego dissolution might exacerbate trauma-related dissociation; the absence of safety signals to that effect in this first dedicated trial is reassuring, but the study was non-randomized, open-label, and the correlation between self-transcendent experience intensity and CAPS-5 improvement, while neurobiologically coherent, is an associational finding from a small, uncontrolled sample and cannot establish a causal or dose-response relationship. Both findings justify, but do not yet substitute for, adequately powered randomized controlled trials in these populations.

Evidence quality and the unblinding problem: The living systematic review and meta-analysis [48] provides the most methodologically rigorous synthesis of the psilocybin depression literature published to date, and its treatment of functional unblinding deserves particular emphasis rather than passing acknowledgment. Because psilocybin’s acute subjective effects are difficult to disguise, the overwhelming majority of participants and, in many trials, raters as well, can correctly identify whether an active drug was administered; this creates the possibility that expectancy effects, rather than (or in addition to) pharmacological action, account for a meaningful proportion of observed symptom improvement. The authors of the living review emphasize that functional unblinding and expectancy effects are likely to inflate observed treatment effects and caution that pooled effect sizes should therefore be interpreted carefully rather than assumed to reflect the true pharmacological efficacy of psilocybin alone [45]—a finding that directly tempers the magnitude of benefit suggested by the pooled effect size alone, and that should be read as a central, load-bearing qualification of the clinical evidence summarized throughout this review rather than as a peripheral caveat. Until psychoactive, psychedelic-naive placebo formulations capable of producing comparable somatic and mild perceptual effects are validated and adopted at scale, the field’s effect size estimates should be treated as an upper bound on true pharmacological efficacy rather than a settled quantity.

At the same time, functional unblinding in psychedelic trials represents a methodological challenge that is not easily resolved by conventional trial design. If the subjective psychological experience itself contributes to therapeutic efficacy, then interventions intended to strengthen blinding by attenuating or masking these experiences may also diminish mechanisms that are potentially integral to treatment response. Consequently, this issue should not be viewed solely as a limitation of trial execution or investigator methodology, but rather as a fundamental challenge inherent to evaluating interventions whose therapeutic effects may arise through an interaction between pharmacological action and psychologically meaningful subjective experiences. Future trial designs will therefore need to balance methodological rigor with preservation of the experiential components that may themselves be clinically relevant.

Safety profile and its limits: Across the trials reviewed, psilocybin has demonstrated an acceptable acute tolerability profile under structured clinical supervision, with transient anxiety, headache, nausea, and dose-dependent elevations in blood pressure and heart rate as the most frequently reported adverse events (Section 4.1 and Section 5.3). Suicidality monitoring remains a standard and necessary component of trial protocols in TRD and Veteran populations, consistent with practice in the COMP001 trial [40]; no completed suicide has been causally attributed to psilocybin administration in any clinical study reviewed here. This safety record, however, applies specifically to the narrow, intensively screened populations enrolled in controlled trials—individuals without personal or first-degree family history of psychotic or bipolar I disorder, without uncontrolled cardiovascular disease, and without active suicidal intent (Section 5.3)—and under conditions of continuous clinical supervision across a multi-hour dosing session. Cumulative safety data beyond the 12-month horizon, data in patients with the comorbid medical and psychiatric complexity typical of real-world psychiatric populations, and data on repeated or maintenance dosing schedules remain limited. The safety profile established to date should therefore be understood as applicable to a circumscribed clinical and demographic envelope, and extrapolation beyond it is not yet supported by the evidence.

Clinical integration challenges: A 2025 consensus statement from the U.S. National Network of Depression Centers cautioned that the pace of scientific discovery, media attention, and commercial interest surrounding psilocybin-assisted therapy risks outpacing the clinical infrastructure required for safe implementation. This review’s findings substantiate that concern in several concrete respects. First, the therapeutic model evaluated in most contemporary clinical trials incorporates a resource-intensive psychotherapeutic scaffold—including preparatory sessions, supervised dosing, and structured integration—which may pose substantial implementation challenges for many healthcare systems. However, it remains uncertain which components of these protocols are essential for achieving optimal therapeutic outcomes, and whether similarly effective but less resource-intensive models could be developed in future clinical practice. A similar implementation challenge has been recognized for other rapid-acting psychiatric interventions, including intranasal esketamine, where successful clinical translation depends on the coordinated integration of psychiatric, psychotherapeutic, and nursing care within standardized multidisciplinary treatment pathways [49]. Second, the pharmacokinetic interaction between psilocybin and concomitant SSRIs/SNRIs (Section 4.1) means that the existing evidence base predominantly reflects psilocybin administered as monotherapy following drug washout, a regimen that maps poorly onto the reality of psychiatric patients who are frequently maintained on long-term antidepressant therapy; standardized protocols for medication management around psilocybin administration do not yet exist. Third, mechanisms to prevent therapeutic boundary violations during extended, non-ordinary states of consciousness, and credentialing standards for the personnel who supervise these sessions, remain under active development rather than settled practice. None of these challenges undermines the pharmacological and clinical signal reviewed in this article. Rather, they highlight uncertainties regarding how best to translate trial-based treatment models into routine clinical practice. Future implementation studies will be essential to determine which elements of current protocols are indispensable for efficacy and safety and which may be adapted to improve scalability, accessibility, and cost-effectiveness without compromising therapeutic benefit.

In addition to the limitations of the available evidence discussed above, several methodological limitations of this review should be acknowledged. First, this study was designed as a narrative review rather than a systematic review; therefore, formal PRISMA reporting guidelines, duplicate study screening, and standardized risk-of-bias assessment tools were not applied. Although predefined eligibility principles were used to enhance transparency, study selection and narrative synthesis remain subject to a degree of author judgment. Furthermore, only publications written in English were included, potentially excluding relevant evidence published in other languages. Finally, while a comprehensive search was conducted across multiple major bibliographic databases, the possibility that relevant studies were not identified cannot be completely excluded.

## 8. Conclusions

Psilocybin has moved from a substance with millennia of ethnobotanical use to a pharmacological candidate supported by a growing, though still methodologically constrained, clinical evidence base. The trials and syntheses reviewed here—anchored by four studies published in 2025–2026—indicate clinically meaningful signals across MDD, TRD, cancer-related existential distress, tobacco and alcohol use disorders, and, more preliminarily, PTSD and severe treatment-resistant depression in Veterans, mediated by a neurobiological mechanism—5-HT2A-dependent neuroplasticity and Default Mode Network modulation—that is pharmacologically distinct from chronic monoaminergic antidepressant action. This evidence remains limited by the unblinding inherent to trials of a subjectively unmistakable agent, by the small, open-label design of the newest indication-expanding studies, and by the narrow, intensively screened populations in which safety has so far been established. Confirmatory randomized controlled trials, durability comparisons against existing maintenance therapy, and credible psychoactive placebo conditions are the immediate priorities; until these are met, psilocybin-assisted therapy is best regarded as a mechanistically distinct and clinically promising, but not yet fully validated, addition to psychiatric practice.

## Data Availability

The original contributions presented in this study are included in the article. Further inquiries can be directed to the corresponding authors.

## Abbreviations

The following abbreviations are used in this manuscript:

AUD	Alcohol Use Disorder
BDNF	Brain-Derived Neurotrophic Factor
CAPS-5	Clinician-Administered PTSD Scale for DSM-5
COMP001/COMP004/COMP360	COMPASS Pathways trails
CYP	Cytochrome P450
CYP2D6/CYP3A4	CYP450 isoform
FDA	Food and Drug Administration
MAO-A	Monoamine Oxidase A
MDD	Major Depressive Disorder
mTOR	mammalian Target Of Rapamycin
PTSD	Post-Traumatic Stress Disorder
QTc	Corrected QT interval
RCT(s)	Randomized Controlled Trial(s)
SNRI(s)	Serotonin-Norepinephrine Reuptake Inhibitor(s)
SSRI(s)	Selective Serotonin Reuptake Inhibitor(s)
TRD	Treatment-Resistant Depression
TrkB	Tropomyosin receptor Kinase B
UGT1A9/UGT1A10	UDP-glucuronosyltransferaze 1A9/1A10
5-HT1A/5-HT1D/5-HT2A/5-HT2B/5-HT2C	Serotoninergic receptors (5-hydroxytryptamine)
